# Leveraging artificial intelligence policy for inclusive and sustainable youth entrepreneurship: micro evidence from China

**DOI:** 10.3389/fpubh.2026.1745563

**Published:** 2026-01-27

**Authors:** Lili Wang

**Affiliations:** Department of Management Science and Engineering, Northeast Petroleum University, Daqing, Heilongjiang, China

**Keywords:** artificial intelligence policy, entrepreneurial environment, relational spending, sustainable development, youth entrepreneurship

## Abstract

Artificial intelligence (AI) policy is increasingly used to align digital transformation with the Sustainable Development Goals by fostering decent work, innovation, and reduced inequality. Youth entrepreneurship is a key channel through which these objectives materialize, yet the micro level pathways linking AI policy to entrepreneurial entry among young adults remain under specified. Using five waves of China Family Panel Studies microdata from 2014 to 2022 and the staggered introduction of AI pilot cities, I estimate a two way fixed effects difference in differences model with rich individual, household, and regional controls. AI pilot status increases the probability of youth entrepreneurship, with baseline effects statistically significant at conventional levels and robust to event study tests of parallel trends, placebo reallocations, alternative time trend controls, and trimming of extreme values. Mechanism analyses show that AI policy operates by reducing relational spending and relaxing credit constraints, as verified by structural equation modeling and multiple mediation tests. Effects are stronger among healthier respondents, those with internet access, and rural residents. This study contributes to the international literature by providing micro-evidence from an emerging economy on how place-based AI policies can function as environmental equalizers. The findings suggest that the observed surge in youth entrepreneurship is structurally motivated by the substitution of digital rules for informal Guanxi and the replacement of physical collateral with digital credit scoring, offering scalable lessons for designing inclusive innovation policies.

## 1 Introduction

Artificial intelligence (AI) policy has become central to aligning digital transformation with the Sustainable Development Goals (1, 2). By shaping data governance, digital infrastructure, standards, and application ecosystems, well designed AI policies can promote decent work and entrepreneurship, strengthen innovation capacity and resilient infrastructure, and reduce inequality of opportunity, directly speaking to SDG 8 (Decent work and economic growth), SDG 9 (Industry, innovation and infrastructure), SDG 10 (Reduced inequalities). Youth entrepreneurship is a vital conduit through which these sustainability objectives are realized in the real economy (3, 4). Young founders convert digital capabilities into new products and services, extend employment margins, and diffuse productivity gains across regions and social groups (5). Understanding how AI policy environments influence entrepreneurial entry among young adults is therefore consequential for an inclusive and sustainable growth agenda (6).

International practice underscores this sustainability link. While the EU and US focus on regulatory sandboxes and commercialization pipelines respectively, Asian economies like Singapore have integrated governance frameworks with SME digitization schemes. These global experiences suggest that AI policy can act as an environmental equalizer, yet specific evidence on localized implementation in emerging economies remains limited (7–10). Across these regimes, evidence from program evaluations and sectoral reports points to shorter time to market, wider diffusion of AI capabilities among smaller firms, and improved access to finance and public procurement. These outcomes are consistent with inclusive growth and sustained innovation capacity and they position AI policy as an environmental equalizer rather than a narrow technology push.

China's AI pilot zone initiative provides a distinctive policy setting with clear origins and design logic that also contributes to the global debate on governing general purpose technologies (11, 12). The pilots build on the 2017 New Generation Artificial Intelligence Development Plan^1^ and on parallel agendas such as Digital China and smart city programs. In 2019, the central government initiated a competitive selection mechanism, inviting qualified municipalities to submit comprehensive proposals for the implementation of AI pilot zones.^2^ Selection typically considered the strength of the local industrial base, research capacity, data resources, and the presence of application scenarios where measurable public value could be demonstrated. The policy rationale combined three aims. First, to accelerate diffusion beyond frontier firms by lowering access costs to data, compute, and engineering support. Second, to institutionalize data governance and ethics practices in real settings, including privacy protection, security review, and model assurance. Third, to build standards, tooling, and public service platforms that reduce coordination failures in fragmented entrepreneurial ecosystems. Beginning in 2019 and expanding in subsequent waves, designated cities committed to integrated investments in digital infrastructure, data governance rules and portals, shared compute and model services, and application portfolios in areas such as mobility, healthcare, manufacturing, and urban services. Crucially, the design of these ‘AI Pilot Zones' creates a quasi-experimental setting. Unlike broad national strategies, the pilots involve distinct, staggered injections of digital infrastructure and policy resources into specific localities. This variation allows for the identification of causal effects on micro-level entrepreneurial behavior, distinguishing the impact of specific policy interventions from general technological trends.

The academic literature on AI and entrepreneurship has advanced along several complementary fronts. At the ecosystem level, AI is framed as a strategic catalyst that couples innovation with sustainability and reweights the salience of entrepreneurial resources with heterogeneous effects across venture types (13, 14). In education, studies show that generative AI supported teaching raises entrepreneurial self-efficacy and intention, particularly where universities provide supportive environments, and that instructors are embedding big data and machine learning into opportunity discovery and evaluation (15–17). In finance and risk management, AI enabled screening and monitoring improve credit assessment and enterprise risk governance, expanding financing access and stimulating local entrepreneurship when banks adopt analytical tools (18, 19). Micro level work grounded in social cognitive and event models finds that AI tools increase digital entrepreneurial intention through perceived desirability, feasibility, and self-efficacy, although technostress can weaken these pathways (20–22). Research on social entrepreneurship indicates that AI and big data enhance transparency and stakeholder engagement, building legitimacy and managerial capability in impact oriented ventures (23). Bibliometric and systematic reviews map the field's intellectual structure and its evolution from early neural network applications to crowdfunding and e health, and they identify the factors shaping AI acceptance and use by entrepreneurs (24, 25).

Despite these advances, important gaps remain. First, most studies examine broad national strategies or generic technology effects rather than targeted, place based policies such as pilot zones that combine resource prioritization, institutional experimentation, and mandated applications (14, 26). Second, youth are rarely analyzed as a distinct population, which leaves the policy to entrepreneurship connection for young adults under specified and limits policy relevance for inclusion agendas (27, 28). Third, prior work seldom foregrounds the sustainability relevant channel in which AI policy improves the entrepreneurial environment by lowering non-productive transaction costs and relaxing structural barriers that perpetuate unequal opportunity, including heavy reliance on relational spending and limited access to collateral based credit (29, 30). Addressing these gaps requires context rich evidence that links concrete AI policy instruments to measurable changes in youth entrepreneurial behavior.

This study responds to that need by using five waves of nationally representative microdata from the China Family Panel Studies for 2014, 2016, 2018, 2020, and 2022, together with the staggered introduction of AI pilot cities summarized in Table 1. The empirical design follows international evaluation practice in industrial and innovation policy by employing a two way fixed effects difference in differences framework with rich individual, household, and regional controls and a comprehensive set of robustness checks. In doing so, the analysis connects a major emerging economy's AI policy experiment to a broader set of global policy experiences and sustainability objectives.

**Table 1:** The pilots of AI.

**Batch**	**Year**	**Province**	**City**
First	2019	Shanghai	Shanghai
	2019	Shandong Province	Jinan City
	2019	Guangdong Province	Shenzhen City
	2019	Shandong Province	Qingdao City
Second	2021	Beijing	Beijing
	2021	Tianjin	Tianjin
	2021	Guangdong Province	Guangzhou City
	2021	Sichuan Province	Chengdu City
	2021	Zhejiang Province	Hangzhou City
Third	2022	Jiangsu Province	Nanjing City
	2022	Hubei Province	Wuhan City
	2022	Hunan Province	Changsha City

The study contributes in three ways. First, it treats the AI pilot zone as an independent policy construct with distinctive features in resource allocation and institutional innovation and examines its concrete effects on entrepreneurial activity, thereby moving beyond generic policy frames. Second, it centers youth as the core population of interest and provides fine grained evidence on how a targeted AI policy relates to their entrepreneurial decisions. Third, it adopts an environmental lens to show how AI policy can improve the fairness and efficiency of the entrepreneurial setting in ways that align with inclusive and sustainable development, offering practice oriented insights for China and for other developing and developed economies.

## 2 Theory and hypotheses

### 2.1 AI policy and youth entrepreneurship

The resource based view argues that sustainable advantage arises when actors access and deploy resources that are valuable, rare, difficult to imitate, and not easily substituted (31). Public policy can reshape the distribution of such resources by converting scarce technological capabilities into usable inputs for specific populations (32). In the context of youth entrepreneurship, AI policy operates as a resource provision and coordination device (33). It lowers the access cost to data, compute, and algorithmic services, standardizes interfaces to digital tools, and connects young founders to complementary assets such as mentoring, market intelligence, and certification. These policy functions directly address early stage constraints that young adults face, including limited analytical capacity, narrow information channels, and weak data support for decision making (34). By building inclusive access platforms and regulating service quality, AI policy turns advanced analytics, digital process tools, and data validation into common capabilities rather than privileges of incumbents. This equalization aligns with sustainable development objectives that emphasize opportunity equality and diffusion of productivity gains across regions and social groups.

AI policy also reorganizes the entrepreneurial ecosystem from a fragmented landscape of isolated services into an integrated system that combines technology and support services (35). This system reduces trial and error costs, lowers perceived risk, and improves the stability of project quality. Critically, it weakens traditional barriers that channel opportunities toward resource rich groups, thereby broadening the feasible set of ventures for young entrepreneurs from diverse backgrounds. On this basis I expect a positive relationship between AI policy exposure and youth entrepreneurial entry.

Therefore, research hypothesis H1 can be obtained:

**H1. AI policy implementation increases the probability that young adults choose entrepreneurship**.

### 2.2 Transmission mechanisms

The effect of AI policy on youth entrepreneurship does not arise solely from generic resource provision. It works through targeted relief of binding constraints that suppress entrepreneurial entry among young adults. Two constraints are central for both inclusion and sustainability. The first is high non-productive transaction cost associated with reliance on informal relations—often referred to as Guanxi in the Chinese context—to obtain permits, information, and partnerships. The second is limited access to finance due to collateral based lending practices that disadvantage young people with thin credit files and few tangible assets.

AI policy reduces the first constraint by digitizing and standardizing public services and market intermediation (36). By digitizing administrative approvals and standardizing market matching, AI policy reduces the necessity of Guanxi. Drawing on the theory of Institutional Voids (37), informal networks often serve as imperfect substitutes for weak market infrastructure. By establishing transparent digital platforms, AI policy reduces the ambiguity that necessitates relational spending, effectively lowering the transaction costs of entry. In many emerging economies, interpersonal connections serve as an informal substitute for weak institutions. AI portals replace this ‘relational tax' with transparent, rule-based digital interfaces, allowing young founders to obtain key inputs through formal channels. Resources can thus be reallocated from costly relationship maintenance to core productive activities.

AI policy alleviates the second constraint by encouraging AI enabled credit assessment and risk management in financial institutions (38). From the perspective of Information Asymmetry theory, traditional lending relies heavily on collateral because lenders lack verifiable information about borrowers. AI-enabled algorithms bridge this gap by processing ‘soft information' (e.g., behavioral footprints and transaction histories) to generate predictive risk profiles. This allows lenders to substitute digital information for physical collateral, directly benefiting youth with ‘thin' credit files (39). AI policy alleviates the second constraint by promoting the adoption of digital credit scoring technologies that utilize alternative data. Traditional lending relies heavily on fixed assets as collateral, disadvantaging youth with ‘thin credit files.' AI-enabled credit scoring translates intangible signals (e.g., technical skills, online behaviors) into measurable risk assessments. This effectively substitutes physical collateral with information capital, relaxing the binding liquidity constraints that typically deter entry for asset-poor entrepreneurs.

Therefore, research hypothesis H2 can be obtained:

**H2. AI policy promotes youth entrepreneurship by reducing spending on relationship maintenance and by relaxing credit constraints**.

**Figure 1:**
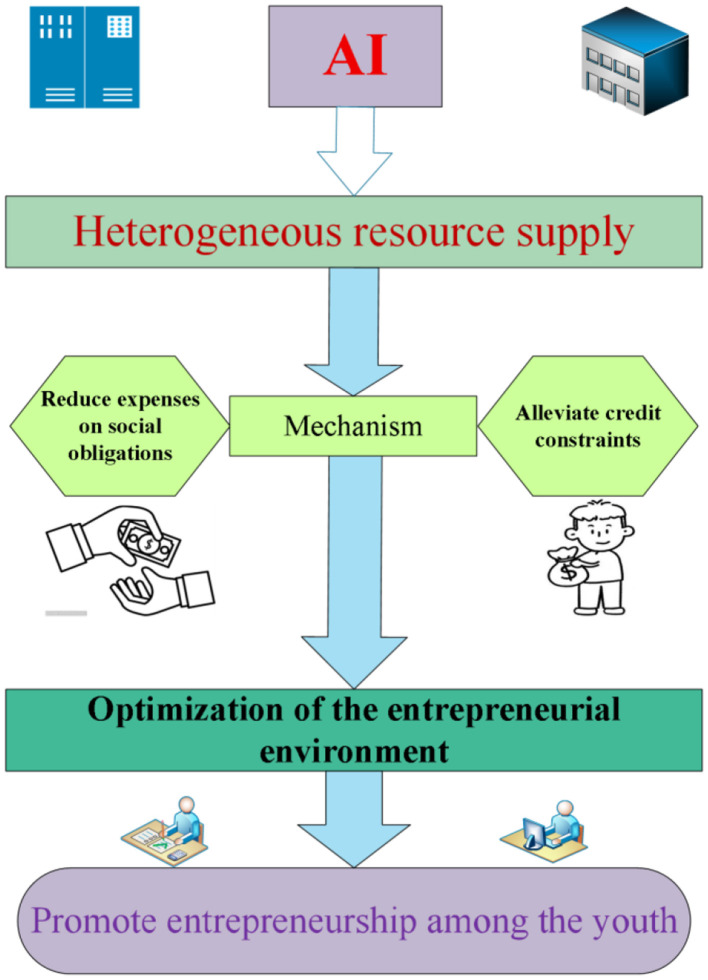
The mechanism by which AI promotes youth entrepreneurship decision making.

Figure 1 presents the conceptual framework that links AI policy to youth entrepreneurial entry through environmental optimization, with the two mechanisms operating as mediators.

## 3 Data and empirical setting

This study leverages a quasi-experimental design based on the staggered implementation of AI pilot zones across China. The specific batches and lists of these pilot cities are detailed in Table 1.

### 3.1 Sample selection and data sources

I draw on the China Family Panel Studies^3^ administered by Peking University. Five survey waves are used: 2014, 2016, 2018, 2020, and 2022. The target population is young adults. I therefore restrict the age range to 18–44 in every observed wave. I remove observations with missing values on the outcome, the policy indicator, or any control variables. I then construct a balanced panel by retaining individuals observed in all five waves after cleaning. I acknowledge potential concerns regarding attrition bias inherent in using a balanced panel over five waves. While this approach ensures I track the same individuals over time, it may exclude those who drop out due to migration or other unobserved factors. However, I mitigate this by employing individual fixed effects to control for time-invariant heterogeneity that might correlate with attrition, and prior studies using CFPS suggest core estimates remain robust across balanced and unbalanced samples.

The final dataset constitutes a panel consisting of a total of 3,794 observations pooled across five survey waves (2014, 2016, 2018, 2020, and 2022). City level covariates are taken from the China City Statistical Yearbook for the corresponding years. I acknowledge that the selection of AI pilot cities is not random; it often correlates with pre-existing industrial and research capacities. This potential endogeneity could threaten validity if pilot cities were already on a different growth trajectory. However, our difference-in-differences design addresses this by controlling for time-invariant city characteristics (via fixed effects) and common time shocks. As long as the trends in entrepreneurship would have been similar in the absence of the policy—a condition supported by our parallel trends test—the non-random selection does not bias the estimator of the treatment effect.

### 3.2 Variables selection

**Explained variable**. Youth entrepreneurship (Job) is coded from the CFPS main work item. Respondents whose main work is private business owner, individual industrial and commercial household, or other self-employment are coded as entrepreneurs with a value of 1. All others are coded 0. This yields a binary indicator that captures entrepreneurial entry status in each wave.

**Explanatory variable**. Exposure to the AI pilot policy is coded at the city by year level and assigned to individuals by their city of residence. For cities designated as pilots, the indicator equals 1 in the years after designation and 0 in pre designation years. For non-pilot cities, the indicator equals 0 in all years.

**Control variables**. Following prior work on AI, entrepreneurship, and inclusive development (40, 41), I include rich individual, household, and regional covariates. Individual characteristics are gender (gender), marital status (married), residence location (resident), self-reported health (health), and education (edu). Gender equals 1 for male and 0 for female. Marital status equals 1 if married and 0 otherwise. Residence equals 1 for rural and 0 for urban. Health is measured on a five point scale where higher values indicate better health. Education is an ordered scale where 0 denotes illiterate or semi-literate, 1 = primary school, 2 = middle school, 3 = high school or equivalent vocational track, 4 = junior college, 5 = bachelor, 6 = master, and 7 = doctorate. Household characteristics are household size (size) and the logs of household income (lnincome) and consumption (lnconsume), both calculated from CFPS items. City level controls are the log of GDP per capita (lnpgdp) and the urbanization rate (urb) for the respondent's city and year.

**Mediating variables**. To study transmission channels that are central to inclusion and sustainability, I construct two mediators. Relational spending is measured as the natural log of social capital outlays (soc) as reported in CFPS. Credit constraint (limit) is a binary indicator that equals 1 if the household reports being credit constrained and 0 if not. Related variable assignment as shown in Table 2.

**Table 2:** Variable definitions and measurements.

**Category**	**Variable name**	**Definition and measurement**
Dependent Variable	Youth Entrepreneurship	Binary variable: equals 1 if the respondent is a private business owner or self-employed; 0 otherwise.
Independent Variable	AI Policy	Binary variable: equals 1 if the city is an AI pilot zone in year $t$ post-implementation; 0 otherwise.
Control Variables	Gender	Dummy variable: 1 = Male, 0 = Female.
	Marital Status	Dummy variable: 1 = Married, 0 = Otherwise.
	Residence	Dummy variable: 1 = Rural, 0 = Urban.
	Health Status	Self-reported health score on a 1–5 scale (higher values indicate better health).
	Education	Ordered scale from 0 (illiterate) to 7 (doctorate).
	Household Size	Total number of family members.
	Household Income	The natural logarithm of total household income.
	Household Consumption	The natural logarithm of total household consumption.
	GDP per capita	The natural logarithm of the city's GDP per capita.
	Urbanization	The ratio of urban population to total population in the city.
Mediators	Relational Spending	The natural logarithm of household expenditures on social relations (Guanxi) and gifts.
	Credit Constraint	Binary variable: equals 1 if the household faces credit rejection or discouragement; 0 otherwise.

### 3.3 Model specification

Building on the theoretical framework, I estimate a two way fixed effects difference in differences model to identify the effect of AI pilot exposure on youth entrepreneurial entry. The baseline specification is


$$\begin{array}{l} \text{JO}{\text{B}}_{\text{i},\text{t}}=\text{α}+\text{βA}{\text{I}}_{\text{i},\text{t}}+\text{γContro}{\text{l}}_{\text{i},\text{t}}+{\text{μ}}_{\text{i}}+{\text{δ}}_{\text{t}}+{\text{ε}}_{\text{i},\text{t}} \end{array}$$ (1)

Here *i* indexes individuals and *t* indexes survey years. The dependent variable *JOB*_*i, t*_ is a binary indicator of youth entrepreneurship coded from the CFPS main work item. The key explanatory variable *AI*_*i, t*_ indicates whether the respondent's city of residence *i* is designated as an AI pilot in year ttt after the adoption year. The vector *Control*_*i, t*_ collects individual, household, and city level controls defined above. The term μ_*i*_ captures time invariant individual heterogeneity and δ_*t*_ captures common shocks by year. The disturbance ε_*i, t*_ is idiosyncratic. I report heteroskedasticity robust standard errors clustered at the individual level to address serial correlation within persons. Under parallel trends, α is a constant term, the coefficient β identifies the average change in the probability of entrepreneurial entry associated with AI pilot exposure, conditional on covariates and fixed effects.

Descriptive statistics for all variables in the balanced panel are reported in Table 3.

**Table 3:** Descriptive statistics.

**Type**	**Variables**	**Count**	**Mean**	**Sd**	**Min**	**Max**
Explained variable	Job	3,794	0.128	0.335	0.000	1.000
Explanatory variable	AI	3,794	0.001	0.023	0.000	1.000
Control variables	Health	3,794	3.168	1.101	1.000	5.000
	Edu	3,794	2.610	1.496	0.000	9.000
	Gender	3,794	0.544	0.498	0.000	1.000
	Married	3,794	0.886	0.318	0.000	1.000
	Resident	3,794	0.777	0.416	0.000	1.000
	Size	3,794	3.101	1.411	1.000	10.000
	lnconsume	3,794	11.128	0.816	8.013	15.458
	lnincome	3,794	10.831	1.412	0.000	15.944
	lnpgdp	3,794	10.852	0.434	10.131	12.155
	Urb	3,794	0.591	0.115	0.374	0.893
Mediating variables	Soc	3,794	7.449	2.035	0.000	12.206
	Limit	3,794	0.361	0.480	0.000	1.000

## 4 Results

### 4.1 Baseline estimates

Table 4 reports the two way fixed effects estimates linking AI pilot exposure to the probability of youth entrepreneurship. In Model 1, which includes individual and year fixed effects but no additional controls, the coefficient on the AI indicator is 0.137 with a robust standard error of 0.012, significant at the one percent level. In Model 2, which adds the full set of individual, household, and city covariates, the coefficient rises to 0.484 with a robust standard error of 0.079, again significant at the one percent level. Given the binary outcome, these coefficients can be read as percentage point changes in entry probability. The controlled estimate implies an increase of 48.4 percentage points for young adults residing in pilot cities relative to comparable peers in non-pilot cities. This substantial magnitude reflects the ‘big push' nature of the AI pilot policy. These zones do not merely offer a single subsidy but implement a comprehensive ecosystem overhaul—simultaneously lowering informational, financial, and administrative barriers. For young potential entrepreneurs facing high initial constraints, this multi-dimensional relief can trigger a non-linear ‘tipping point' in occupational choice, explaining the large estimated effect.

**Table 4:** Benchmark regression results.

**Variables**	**Model 1**	**Model 2**
AI	0.137^***^ (0.012)	0.484^***^ (0.079)
Constant	1.366^***^ (0.107)	2.159 (9.905)
*N*	3,794	3,794
idfix	Yes	Yes
Yearfix	Yes	Yes
Control variables	No	Yes

The figures in parentheses are the robust standard errors of clustering to the person level.

^***^ indicate significance at the 1% levels, ^***^p < 0.01.

The increase in magnitude from Model 1 to Model 2 suggests negative confounding in the uncontrolled specification, where omitted factors that depress entrepreneurship are more prevalent in pilot locations and are absorbed once controls are included. Taken together, the baseline results provide clear support for H1 that AI policy promotes youth entrepreneurial entry.

### 4.2 Robustness checks

#### 4.2.1 Parallel trends

**Figure 2:**
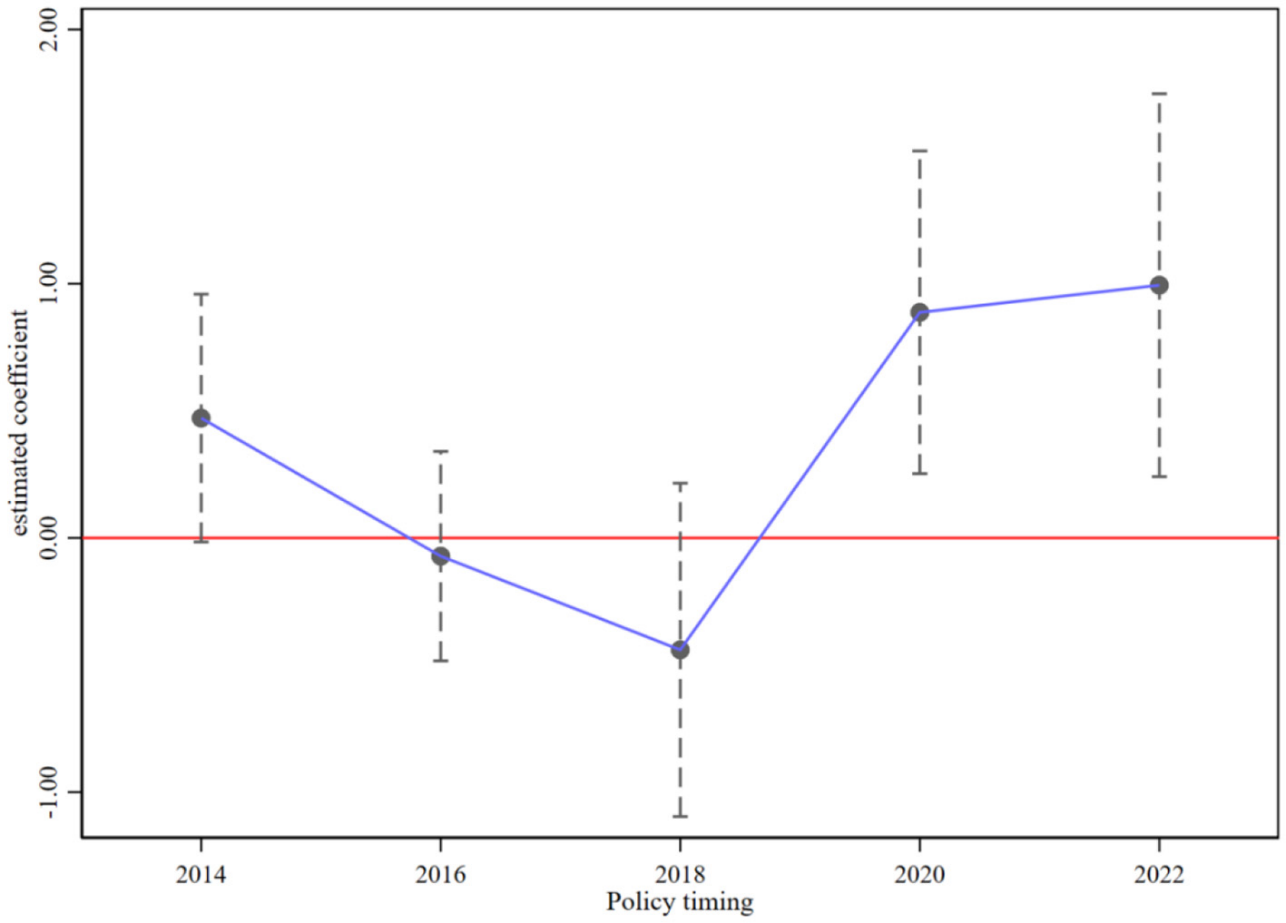
Parallel trend test.

Figure 2 presents the event study coefficients relative to policy adoption. The coefficients for the periods leading up to the policy implementation fluctuate around zero and are statistically insignificant. This lack of significance confirms the absence of systematic divergent trends between pilot and non-pilot cities prior to the intervention, thereby satisfying the parallel trends assumption required for causal identification. After adoption, the relative time coefficients become positive and their confidence intervals exclude zero. These dynamics support the parallel trends assumption and indicate that the treatment effect emerges only after policy implementation.

#### 4.2.2 Placebo assignments

**Figure 3:**
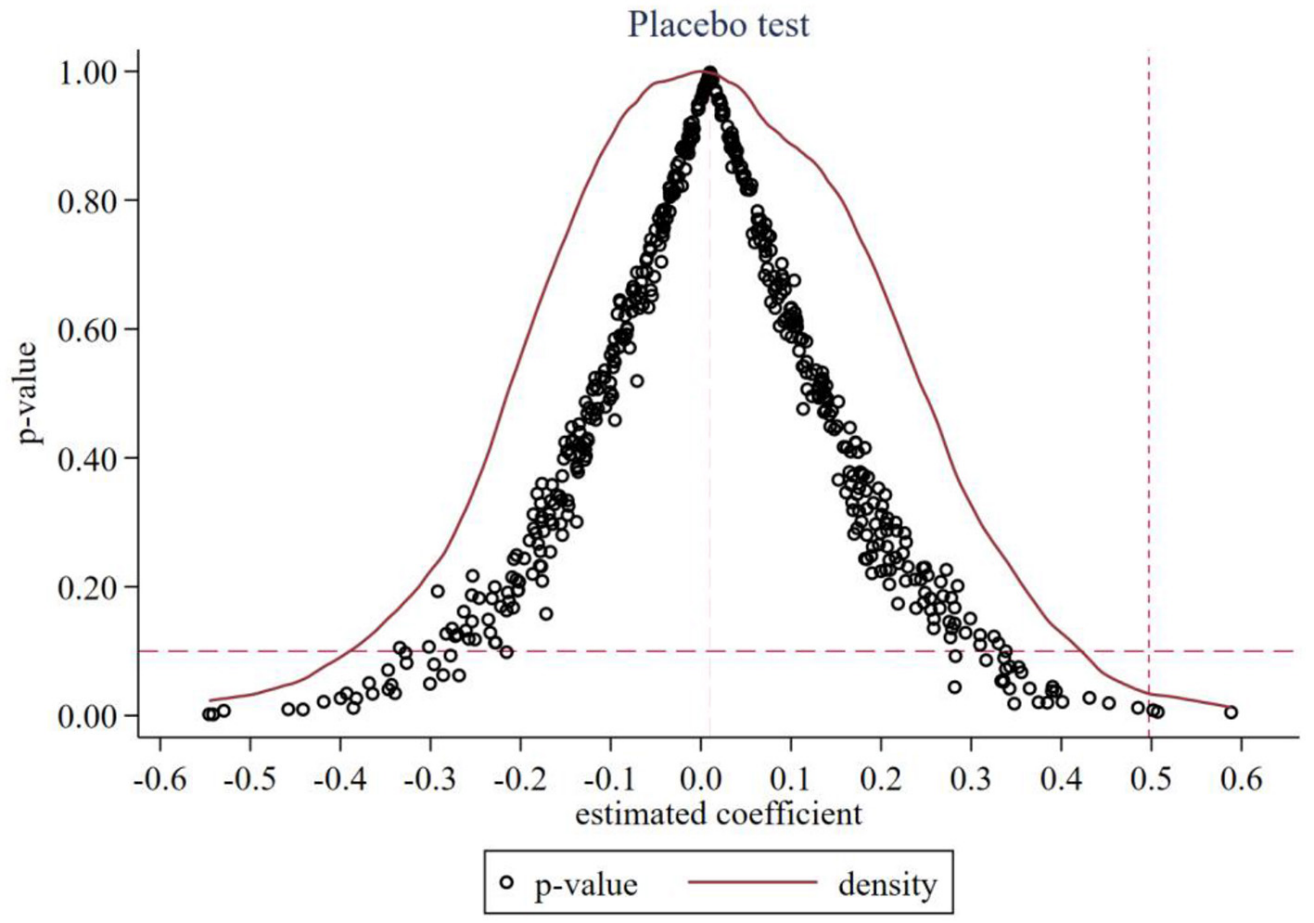
Results of placebo test.

I conduct placebo tests by randomly assigning the policy to cities and re estimating the baseline model 500 times (Figure 3). The resulting distribution of placebo coefficients centers tightly around zero and most *p*-values exceed 0.10. The true estimate lies in the positive tail and is clearly separated from the placebo mass. The fact that our estimated coefficient is located at the extreme end of the distribution implies that the probability of observing such a result purely by chance is negligible. This statistically validates that the observed effect is driven by the specific timing and location of the AI policy, rather than by random noise or unobserved confounding factors common to the placebo simulations. This pattern suggests that the baseline effect is not an artifact of chance assignment or of latent confounders that would generate spurious correlations.

In our context, the placebo results lend credibility to the interpretation that AI policy exposure, rather than general digital trends, is responsible for the estimated increase in youth entrepreneurship. This interpretation is consistent with sectoral evidence that targeted AI initiatives, such as financial digitization, produce localized entrepreneurial responses over and above background trends (42, 43).

#### 4.2.3 Alternative time trend controls

To probe sensitivity to differential city dynamics, I replace city level controls with interactions between city characteristics and linear time trends and then with quadratic time trends. As shown in Table 5, the AI policy coefficient remains positive and statistically significant at the 10 percent level, with magnitudes close to the baseline. This indicates that unobserved city specific trends correlated with observable characteristics are unlikely to drive the results. Allowing for city specific trends addresses a common critique of difference in differences in spatial policy evaluations (44).

**Table 5:** Propensity match score.

**Variables**	**Model 3**	**Model 4**
AI	0.492^*^ (0.283)	0.386^*^ (0.232)
Constant	2.260 (9.908)	3.448 (9.985)
*N*	3,794	3,794
idfix	Yes	Yes
Yearfix	Yes	Yes
Control variables	Yes	Yes

The robust standard errors clustered at the person level are in parentheses.

^*^ indicate significance at the 10% levels, ^*^p < 0.1.

#### 4.2.4 Trimming extreme observations

Difference in differences estimates can be sensitive to leverage from extreme values. I therefore trim core variables at the 90th percentile and re estimate the models. Table 6 shows that the estimates with and without controls are close to baseline magnitudes and significance levels, which suggests that the results are not driven by outliers in either covariates or outcomes. Robustness to trimming is consistent with the notion that AI policy effects diffuse through broad based improvements in service standardization and credit analytics rather than being concentrated in a few atypical cities (45).

**Table 6:** Eliminate outliers.

**Variables**	**Model 5**	**Model 6**
AI	0.139^**^ (0.011)	0.386^*^ (0.231)
Constant	1.466^***^ (0.097)	5.544 (9.642)
*N*	3,794	3,794
idfix	Yes	Yes
Yearfix	Yes	Yes
Control variables	No	Yes

The robust standard errors clustered at the person level are in parentheses.

^*^, ^**^, and ^***^ indicate significance at the 10%, 5%, and 1% levels, respectively. ^*^p < 0.1, ^**^p < 0.05, ^***^p < 0.01.

#### 4.2.5 Heterogeneous treatment weights

I implement the decomposition proposed by De Chaisemartin and D'Haultfoeuille (27) to assess the weight structure of the two way fixed effects estimator. As highlighted by De Chaisemartin and D'Haultfoeuille (27), in staggered difference-in-differences designs, the standard Two-Way Fixed Effects (TWFE) estimator represents a weighted average of treatment effects. If a significant portion of these weights is negative, the estimated coefficient may have the opposite sign of the true average treatment effect. Our diagnostic reveals that less than 1% of the weights are negative. This minimal presence confirms that the heterogeneity in treatment timing does not distort our pooled estimates.

#### 4.2.6 Alternative estimations

Given that the dependent variable is binary, I also re-estimated the baseline model using Logit (Model 7) and Probit (Model 8) specifications to validate the linear probability model. The results showed that the marginal effects were consistent with the baseline estimates in terms of sign and statistical significance (Table 7), further supporting the robustness of our findings.

**Table 7:** Alternative estimations.

**Variables**	**Model 7**	**Model 8**
AI	0.476^***^ (0.082)	0.481^***^ (0.080)
Constant	3.454 (15.848)	2.112 (9.885)
*N*	3,794	3,794
idfix	Yes	Yes
Yearfix	Yes	Yes
Control variables	Yes	Yes

The robust standard errors clustered at the person level are in parentheses.

^***^ indicate significance at the 1% levels, ^***^p < 0.01.

#### 4.2.7 Sub-sample analysis

Restricting to Pilot Provinces One potential concern with the DID identification is selection bias: AI pilot cities (e.g., Shanghai, Shenzhen) are often located in economically developed provinces, while control cities may be in underdeveloped inland regions. This regional imbalance could confound the policy effect with pre-existing economic trends. To mitigate this, I conduct a sub-sample analysis by restricting the sample to only include provinces that host at least one AI pilot city. This approach ensures that the control group (non-pilot cities) is drawn from the same broader regional economic environment as the treatment group, making them more comparable counterfactuals. As presented in Table 8, the coefficient of the AI policy variable remains positive and statistically significant. The magnitude is consistent with the baseline results, confirming that the observed surge in youth entrepreneurship is driven by the AI policy intervention itself rather than by unobserved heterogeneity between developed and underdeveloped provinces.

**Table 8:** Sub-sample regression results.

**Variables**	**Model 9**	**Model 10**
AI	0.142^***^ (0.015)	0.465^***^ (0.081)
Constant	1.288^***^ (0.112)	1.954 (8.762)
*N*	2,480	2,480
idfix	Yes	Yes
Yearfix	Yes	Yes
Control variables	No	Yes

The robust standard errors clustered at the person level are in parentheses.

^***^ indicate significance at the 1% levels, ^***^p < 0.01.

### 4.3 Mechanism evidence

Building on the theoretical framework, I evaluate two mediators that are central to inclusive and sustainable development: reductions in relational spending and alleviation of credit constraints. Structural equation models with individual and year fixed effects indicate statistically significant indirect effects for both channels. For relational spending as shown in Tables 9, 10, the estimated indirect effect is −0.004 with *p*-values around 0.007 to 0.008 across Delta, Sobel, and Monte Carlo tests. For credit constraints, the estimated indirect effect is −0.003 with *p*-values around 0.029–0.033. Negative signs indicate that AI policy reduces relational outlays and the incidence of binding credit constraints, which in turn increases the probability of entrepreneurial entry among young adults. Therefore, H2 is proven. Meanwhile, in terms of model validity, the structural equation modeling results demonstrate a satisfactory model fit, with a Comparative Fit Index (CFI) of 0.91 and a Root Mean Square Error of Approximation (RMSEA) of 0.048, validating the hypothesized paths.

**Table 9:** The results of the “soc” mediating effect test.

**Estimated value**	**Delta**	**Sobel**	**Monte carlo**
Indirect effect	−0.004	−0.004	−0.004
Std. Err.	0.002	0.002	0.002
*z*-value	−2.687	−2.689	−2.658
*p*-value	0.007	0.007	0.008
[95% conf. interval]	[−0.007, −0.001]	[−0.007, −0.001]	[−0.007, −0.001]

**Table 10:** The results of the “limit” mediating effect test.

**Estimated value**	**Delta**	**Sobel**	**Monte carlo**
Indirect effect	−0.003	−0.003	−0.003
Std. Err.	0.001	0.001	0.001
*z*-value	−2.177	−2.179	−2.131
*p*-value	0.029	0.029	0.033
[95% conf. interval]	[−0.005, −0.000]	[−0.005, −0.000]	[−0.005, −0.000]

These mediation results map closely to international evidence that AI enhances information production and credit risk assessment, which expands financial inclusion in emerging economies, improves peer to peer screening quality, and supports ethical and capability building dimensions of entrepreneurship education that reduce reliance on informal networking (46, 47). They also connect to findings that AI enabled risk management fosters entrepreneurship and performance in financial institutions and to evidence on the role of AI learning in strengthening student entrepreneurs' outcomes (48). The mechanisms therefore provide a micro level bridge between technology policy and the SDGs agenda on decent work and reduced inequality.

Based on this, H2 has been proven.

### 4.4 Heterogeneity

**Figure 4:**
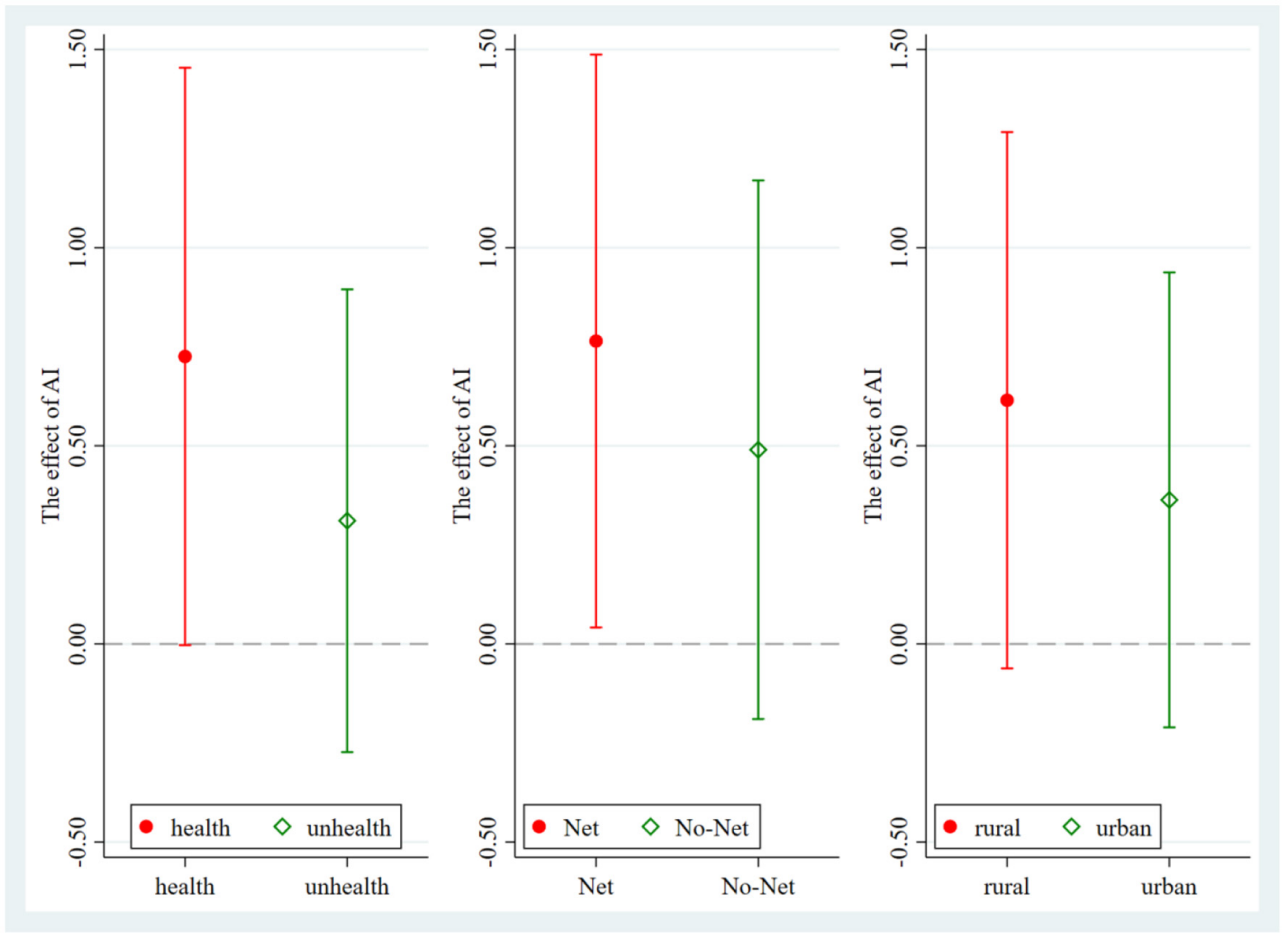
Heterogeneity test results.

I examine heterogeneous impacts along three dimensions that are salient for inclusive and sustainable development: health status, digital access, and urban–rural residence. Results are summarized in Table 11 and visualized in Figure 4.

**Table 11:** Results of heterogeneity test.

**Variables**	**Model 11**	**Model 12**	**Model 13**	**Model 14**	**Model 15**	**Model 16**
	**Health**	**Unhealth**	**Net**	**No-Net**	**Rural**	**Urban**
AI	0.725^*^ (0.372)	0.311 (0.297)	0.764^**^ (0.368)	0.490 (0.346)	0.615^*^ (0.345)	0.363 (0.293)
constant	2.672 (10.503)	−10.371 (12.672)	23.883 (15.882)	3.365 (14.113)	0.435 (12.612)	11.603 (10.645)
*N*	3,446	348	1,983	1,811	1,998	1,796
idfix	Yes	Yes	Yes	Yes	Yes	Yes
Yearfix	Yes	Yes	Yes	Yes	Yes	Yes
Control variables	Yes	Yes	Yes	Yes	Yes	Yes

The robust standard errors clustered at the person level are in parentheses.

^*^, ^**^ indicate significance at the 10% and 5% levels, respectively, ^*^p < 0.1, ^**^p < 0.05.

#### 4.4.1 Health heterogeneity

Using the CFPS six waves chronic illness indicator, I split the sample into healthy and unhealthy groups. The AI policy coefficient is positive in both subsamples but statistically significant only for the healthy group. For healthy respondents the estimate is 0.725 with a robust standard error of 0.372, significant at the 10 percent level (Model 11). For respondents reporting chronic illness the estimate is 0.311 with a robust standard error of 0.297 and is not statistically significant (Model 12), likely due to the smaller sample size and lower statistical power of this subgroup. This pattern suggests that translating policy enabled improvements in information flow, process management, and decision quality into entrepreneurial action requires sufficient physical and cognitive bandwidth to learn tools, implement decisions, and sustain effort.

The result is consistent with the complementarity view that AI augments human capital and that benefits accrue where absorptive capacity is higher (49). It echoes education evidence that generative AI raises entrepreneurial self-efficacy when learners can invest in capability building, and it aligns with studies that link AI related learning to stronger entrepreneurial outcomes among students (50).

#### 4.4.2 Digital access heterogeneity

I proxy digital access by internet use. Effects are larger and significant for internet users, with an estimate of 0.764 and a robust standard error of 0.368, significant at the five percent level (Model 13). For non-users the estimate is 0.490 with a robust standard error of 0.346 and is not statistically significant (Model 14). The contrast indicates that connectivity is a precondition for converting AI policy into usable services such as digital public portals and data driven market matching, which lower entry barriers and improve the quality of entrepreneurial decisions.

#### 4.4.3 Urban–rural heterogeneity

I split the sample by residence. Coefficients are positive in both groups but larger and statistically significant in rural areas. The rural estimate is 0.615 with a robust standard error of 0.345, significant at the 10% level (Model 15). The urban estimate is 0.363 with a robust standard error of 0.293 and is not statistically significant (Model 16). The larger rural effect is consistent with higher marginal returns where initial constraints on information, intermediation, and service quality are more severe. Digital public services and AI enabled intermediation relax precisely those structural barriers that bind more tightly in rural contexts, thereby yielding greater relative gains for rural youth.

The result aligns with calls to study place based AI policies and their distributional consequences and supports the framing of AI policy as an environmental equalizer that narrows spatial opportunity gaps central to SDG objectives on decent work and reduced inequalities (51). It also resonates with evidence that targeted AI adoption in finance can stimulate local entrepreneurship where credit frictions are more binding (52, 53).

## 5 Discussion and conclusions

Using five CFPS waves from 2014 to 2022 and the staggered rollout of AI pilot cities, I find that AI policy significantly increases the probability that young adults choose entrepreneurship: The baseline difference in differences estimates are precise and stable across specifications, with the policy coefficient rising when rich controls are added, which indicates that unobserved disadvantage in pilot locations is absorbed once individual, household, and city characteristics are accounted for. This provides clear support for positions AI policy as an environmental equalizer that aligns innovation with opportunity creation for youth. These findings are consistent with syntheses that link AI to entrepreneurial dynamism and inclusive growth in supportive institutional settings (54).

Moreover, mechanism analyses show that AI policy operates through two sustainability relevant channels. First, it reduces non-productive relational spending by digitizing and standardizing key public services and market intermediation. Second, it relaxes credit constraints by enabling more informative screening and monitoring in finance. Structural equation models document statistically significant indirect effects for both mediators. These channels connect technology policy to the Sustainable Development Goals on decent work, industry innovation, and reduced inequalities and echo evidence that AI enabled risk analytics expands financial inclusion and venture activity (54).

Finally, heterogeneity analyses reveal that effects are stronger for healthier youth, for those with internet access, and for rural residents. This pattern is consistent with the view that AI augments human and digital capabilities and yields higher marginal gains where initial constraints on information and intermediation are most binding. It reinforces the interpretation of AI policy as an environmental equalizer whose inclusive benefits depend on complementary investments in access and capability, a theme emphasized in international practice and the research frontier on place based AI policies (55).

### 5.1 Policy implications

First, governments should leverage AI-driven diagnostic algorithms to upgrade entrepreneurship support services. Instead of merely digitizing application forms, policymakers should build intelligent service stacks that use predictive models to automatically match young entrepreneurs with suitable policy incentives based on their profiles. This approach replaces manual eligibility screening with algorithmic diagnostics, thereby reducing administrative friction and non-productive time costs for applicants.

Second, financial regulators should encourage the adoption of machine learning-based credit scoring models to broaden access to finance. Traditional collateral-based lending systematically excludes youth with “thin” credit files. By integrating non-traditional signals—such as digital transaction footprints and behavioral data—into AI risk assessment models, lenders can accurately evaluate creditworthiness without physical collateral. This shift from asset-based to data-based lending is crucial for relaxing the binding liquidity constraints facing young founders.

Third, support measures must be tailored to heterogeneous needs. For rural youth, AI-enabled remote mentoring and market-matching platforms can bridge the spatial divide. For those with health limitations, virtual incubation services ensure that physical constraints do not preclude entrepreneurial entry. Finally, a robust governance framework—incorporating “Explainable AI” (XAI) and algorithmic fairness audits—is essential to ensure that automated decision-making does not reinforce existing inequalities.

Fourth, institutionalize multi stakeholder governance with strong safeguards and continuous evaluation. Establish coordination mechanisms that link technology, labor, finance, taxation and education agencies with industry associations, universities and civil society. Encourage large AI firms to open technical resources through shared platforms and voucher schemes for compute, application programming interfaces and training, and to co run project studios with universities that translate research into venture ready products. Address potential risks by establishing ‘Explainable AI' (XAI) auditing mechanisms. Governance frameworks must ensure that automated approvals do not systematically disadvantage vulnerable groups through algorithmic bias. Privacy protection and clear documentation should be mandated for all AI systems used in credit and public intermediation. Implement specific Key Performance Indicators (KPIs) to monitor success beyond simple entry rates. Recommended KPIs include the ‘Credit Conversion Rate' (percentage of applicants without collateral receiving loans), ‘Digital Service Usage Rate' among rural youth, and the ‘Survival Rate' of AI-supported ventures over 3 years, including youth entry rates, survival, access to formal credit, usage of digital public services and distribution across health, access and location strata. Where feasible, embed randomized or staggered rollouts to enable credible impact assessment and course correction. This governance architecture ties innovation to accountability and ensures that AI policy advances the Sustainable Development Goals on decent work, industry innovation and reduced inequalities.

### 5.2 Limitations and future research

While this study provides robust micro-evidence, several limitations should be noted. First, regarding the identification strategy, I acknowledge that the selection of AI pilot cities is not random; these zones are often established in regions with stronger pre-existing industrial bases. While I employed a Difference-in-Differences (DID) design with comprehensive city fixed effects to control for time-invariant heterogeneity, and the parallel trend tests support the validity of the control group, unobserved time-varying shocks correlated with pilot designation could still theoretically pose identification challenges. Future research could explore natural experiments or instrumental variable strategies to further isolate causal effects from regional economic trends.

Second, the analysis relies on a balanced panel dataset from the CFPS. Although this allows for tracking individual-level changes, the sample size and the restriction to a balanced panel may introduce attrition bias if individuals who drop out of the survey differ systematically from those who remain. Future research utilizing administrative registration data with full population coverage would offer higher precision.

Third, the transmission mechanisms—relational spending and credit constraints—are measured using survey proxies. Entrepreneurial status is measured as a binary main work indicator, which may under-capture emerging forms of informal digital gig work. Future studies could benefit from cooperating with banks or digital platforms to access granular data on loan applications and approval logs, providing more direct evidence of how AI alters credit allocation mechanics.

## Data Availability

The original contributions presented in the study are included in the article/supplementary material, further inquiries can be directed to the corresponding author.

## Footnotes

1 Generation Artificial Intelligence Development Plan: https://www.gov.cn/zhengce/content/2017-07/20/content_5211996.htm.

2 AI pilot zones: https://www.gov.cn/zhengce/zhengceku/2019-12/03/content_5457884.htm.

3 CFPS: https://www.isss.pku.edu.cn/cfps/.
